# Simulation and Experimental Investigation of the Vacuum-Enhanced Direct Membrane Distillation Driven by a Low-Grade Heat Source

**DOI:** 10.3390/membranes12090842

**Published:** 2022-08-29

**Authors:** Qingfen Ma, Liang Tong, Chengpeng Wang, Guangfu Cao, Hui Lu, Jingru Li, Xuejin Liu, Xin Feng, Zhongye Wu

**Affiliations:** 1 College of Mechanical and Electrical Engineering, Hainan University, Haikou 570228, China; 2 The Institute of Seawater Desalination & Multipurpose Utilization, MNR, No. 55, Hanghai Road, Tianjin 300192, China; 3 Institute of Environment and Plant Protection, Chinese Academy of Tropical Agriculture Sciences, Haikou 571101, China

**Keywords:** desalination, VEDCMD, transmembrane mass and heat transfer

## Abstract

Vacuum-enhanced direct contact membrane distillation (VEDCMD) has been proven experimentally to improve the permeate flux, compared with direct contact membrane distillation (DCMD). However, the theoretical mechanism for its transmembrane transfer process has not been revealed sufficiently. In this paper, with full consideration of the different driving forces of diffusion and Poiseuille flow under the vacuum enhancing condition, a theoretical transmembrane model for mass and heat transfer in VEDCMD is proposed. The CFD model and experimental platform are established to verify the theoretical model. The simulated results agree with the experimental data well, and nearly 200% improvement of the permeate flux is obtained when the permeate pressure drops to 30 kPa. The flow fields of the flow along the membrane surface are obtained and analyzed, with good consistency in the variation of the permeate flux. Since all the parameters of the proposed model are independent of the operating condition, the model is much easier for use and has better adaptability to fluctuating operating conditions.

## 1. Introduction

Membrane distillation (MD) is a membrane-based separation technology [1], including direct contact membrane distillation (DCMD), vacuum membrane distillation (VMD), swept gas membrane distillation (SGMD), and air gap membrane distillation (AGMD) [2]. Compared with other MD processes, DCMD is applied much more widely due to its advantages, such as simple installation and operation and no requirement for an external condenser [3]. However, the high energy consumption of DCMD is not only conflicting with the fossil fuel shortage but also detrimental to the environment. Thus, DCMD driven by clean or low-grade heat sources such as solar energy, geothermal energy, and recovery of waste heat has attracted widespread attention. Bamasag et al. [4] proposed a DCMD system that used solar thermal energy for desalination. Enhanced by the solar radiation, the permeate flux of DCMD was improved about 17%. Lokare et al. [5] utilized the waste heat from natural gas compressor stations as the heat source of DCMD, effectively concentrating the high salinity water, regardless of the initial salinity.

Whether the clean or low-grade heat is utilized effectively in the DCMD can be assessed by the freshwater production rate per unit of heat input. Given the same conditions of heat input, membranes, and the fluid flow, the permeate flux is a widely accepted, effective, and available index to evaluate heat utilization efficiency. The higher the permeate flux, the more efficient and effective the water treatment, and the greater the heat utilization efficiency. Thus, plenty of research has been carried out to improve the permeate flux of DCMD, including improving membrane materials [6,7,8,9,10,11,12,13,14], upgrading membrane modules [15,16,17,18], optimizing operating conditions (e.g., increasing the feed-side temperature [19,20,21], and decreasing the permeate-side pressure [22,23,24,25], etc.). Enhancing the vacuum degree of the permeate side has been proven experimentally to improve the permeate flux, especially when there is a limitation for the feed temperature and the membrane material and modules are determined. Cath et al. [22] found in their experiments that the flux of vacuum-enhanced direct contact membrane distillation (VEDCMD) with permeate pressure of 550 mbar was increased by 84% compared to the DCMD with permeate pressure of 1080 mbar under the same operating temperatures. In the experiments of Plattner et al. [25], the feed and permeate side temperature of VEDCMD were 55 °C and 25 °C, respectively. When the permeate pressure decreased from 1000 mbar to 300 mbar, the permeate flux was enhanced by 42%.

As mentioned above, the flux enhancement of VEDCMD has been verified experimentally, but the research on the transmembrane transfer model of VEDCMD is still inadequate. Schofield et al. [26] introduced a dimensionless coefficient *α* into the classical mass transfer model of DCMD to fit the measured fluxes of VEDCMD. Although the classical transfer patterns like diffusion and the Poiseuille flow were both included in the model, all the transfer processes were assumed to be driven only by the vapor pressure difference between the two ends of the micro-pore of membrane, while the driving force caused by the total pressure difference between the feed and permeate side was not considered. Similarly, Naidu et al. [27] developed their mass transfer model based on the model of [26], and the driving force of the transmembrane mass transfer only considered the pressure difference of two membrane surfaces. Since the flux enhancement of VEDCMD compared to the traditional DCMD is closely related to the total pressure difference between the feed and the permeate side, the corresponding driving force should also be properly included in building the mass transfer model after accurately analyzing all the driving forces and their relationships with the dominant mass transfer patterns. As far as the authors know, currently, no published articles have reported the related research results.

In this paper, on the basis of accurately analyzing the driving force for each transmembrane mass transfer pattern of VEDCMD, a theoretical transmembrane transfer model is developed, based on which a computational fluid dynamics (CFD) model for a plate type VEDCMD is established to predict the permeate flux and reveal the distributions of important parameters along the membrane surface, which are hardly obtained through experiments. Finally, to verify the proposed theoretical model and the CFD simulation results, an experimental platform is built, and the permeate fluxes are measured and compared with the simulation results.

## 2. Theoretical and CFD Model

### 2.1. Theoretical Model

#### 2.1.1. Mass Transfer

For the transmembrane mass transfer of DCMD, the only driving force is the vapor pressure difference between the feed and the permeate side. The permeate flux can be calculated by Equation (1) [28].

(1) $$J_{M}=K_{M}\left(p_{f,W}-p_{p,W}\right)$$

where *p*_f,W_ (Pa) and *p*_p,W_ (Pa) are the partial pressures of water vapor on the feed side and permeate side, respectively. *K*_M_ is the mass transfer coefficient. The subscripts “f” and “p” indicate parameters at the feed and permeate side, respectively. The subscripts “W” and “M” indicate the parameters in the flow channel and on the membrane surface, respectively. *p*_f,W_ (Pa) can be calculated according to Equation (2), where *γ*_w_ is the activity coefficient of water as shown in Equation (3) [28]. The subscripts “w” indicate the parameter of water. *X*_NaCl_ is the molar fraction of sodium chloride in feed solution as calculated by Equation (4). The saturation vapor pressure of pure water $p_{v}^{S}$ (*T*) (Pa) at different temperatures *T* (K) can be obtained by the Antoine Equation (5).

(2) $$p_{f,W}=\left(1-X_{\mathrm{NaCl}}\right)p_{v}^{S}\left(T_{f,W}\right)\gamma_{w}$$

(3) $$\gamma_{w}=1-0.5X_{\mathrm{NaCl}}-10X_{\mathrm{NaCl}}{}^{2}$$

(4) $$X_{\mathrm{NaCl}}=\frac{W_{\mathrm{NaCl}}}{M_{\mathrm{NaCl}}}/\left(\frac{W_{\mathrm{NaCl}}}{M_{\mathrm{NaCl}}}+\frac{W_{w}}{M_{w}}\right)$$

(5) $$p_{v}^{S}(T)=23.1964-\frac{3816.44}{\;T-46.13}$$

The dust gas model (DGM) proposed by Masson et al. [29] was widely accepted to characterize the transmembrane mass transfer mechanism of MD. Based on the DGM model, Schofield et al. [30] classified the transmembrane mass transfer patterns as follows: the Knudsen diffusion, the molecular diffusion, the Knudsen-Molecular diffusion, and the Poiseuille flow. The Knudsen number *K*_n_ can be calculated by Equation (6).

(6) $$K_{n}=\frac{\lambda }{d}$$

where *λ* (μm) refers to the mean molecular free path of water vapor, and *d* (μm) is the mean diameter of the membrane pores. Because the air stays in the membrane pores, and is hardly soluble in the external inflow, an air-vapor co-existence environment is formed in the pore. Thus, the mean molecular free path of the gas in the pore can be calculated by Equation (7) [28].

(7) $$\lambda_{w-a}=\frac{K_{B}T_{m}}{\pi {\left(\frac{\beta_{w}+\beta_{a}}{2}\right)}^{2}p\sqrt{1+\left(\frac{M_{w}}{M_{a}}\right)}}$$

where *K*_B_ is the Boltzmann constant (1.381 × 10^−23^ J·mol^−1^·K^−1^) and *T*_m_ (K) is the average temperature of both sides of the membrane. The subscript “*a*” indicates the parameter of air. *β_a_* (2.641 × 10^−4^ μm) and *β_w_* (3.711 × 10^−4^ μm) are the collision diameters of air and water molecules, respectively. *p* (Pa) is the absolute pressure, and *M*_w_ and *M_a_* are the molecular weight of water and air, respectively. According to the Knudsen number, the transmembrane mass transfer caused by diffusion can be classified into four patterns.

(1) Molecular diffusion

When the mean molecular free path is smaller than the membrane pore size, leading to *K*_n_ > 1, the collision between molecules becomes the main form of diffusion, and the mass transfer coefficient is calculated by Equations (8) and (9) [30]:

(8) $$K_{M}=K_{\mathrm{MD}}=\frac{p_{t}D_{wa}\varepsilon_{h}}{\tau \delta }\frac{M_{w}}{RT_{m}}$$

(9) $$p_{t}D_{wa}=1.895\times 10^{-5}T^{2.072}$$

where *ε*_h_ is the surface porosity, *δ* (mm) is the thickness of the membrane, *p*_t_ is the gas pressure inside the membrane pore space, *D*_w*a*_ (m^2^·s^−1^) is the diffusion coefficient of water in air, *τ* is the membrane tortuosity factor, and *R* (J·mol^−1^·k^−1^) is the gas constant.

(2) Knudsen diffusion

When the mean molecular free path is much larger than the diameter of the membrane pore, i.e., *K*_n_ < 0.01, the pore size will primarily affect the transmembrane motion of molecules. The collision between molecules and the wall of the membrane pore becomes the major obstruction. In this case, the mass transfer coefficient is calculated by Equation (10) [30].

(10) $$K_{M}=K_{K}=\frac{2}{3}\frac{r\varepsilon_{h}}{\tau \delta }{\left(\frac{8M_{w}}{\pi RT_{m}}\right)}^{0.5}$$

where *r* (μm) is the membrane pore radius.

(3) Knudsen-Molecular diffusion

When 0.1 < *K*_n_ < 1, molecular diffusion and Knudsen diffusion both become the primary forms of molecular mass transfer across the membrane. In this case, using the Knudsen-molecular diffusion model, the mass transfer coefficient can be expressed as Equation (11).

(11) $$K_{M}=K_{K-\mathrm{MD}}={\left(\frac{1}{K_{K}}+\frac{1}{K_{\mathrm{MD}}}\right)}^{-1}$$

(4) Poiseuille flow

When the collisional motion between molecules becomes the dominant form, if a macroscopic total pressure gradient difference is generated on both sides of the membrane, the gas inside the pore will diffuse in the direction of the inverse gradient difference of pressure. The mass transfer coefficient associated with Poiseuille flow *K*_PO_ (kg·m^−2^·s^−1^·pa^−1^) can be expressed as Equation (12) [30].

(12) $$K_{\mathrm{PO}}=\frac{1}{8\mu }\frac{r^{2}\varepsilon_{h}}{\tau \delta }\frac{M_{w}p_{f,W}}{RT_{m}}$$

where *μ* (pa·s) is the viscosity of pure vapor at a specific temperature, which can be calculated by Equation (13). *μ*_0_ (pa·s) and *Su* (K) are the kinetic viscosity and Susland constant of the gas at 0 °C, respectively.

(13) $$\mu =\mu_{0}\frac{273+Su}{T+Su}{\left(\frac{T}{273}\right)}^{15}$$

The above mass transfer model has been proven to be a fit for the flux prediction of DCMD, but the Equation (1) is not applicable to the VEDCMD, for the influence of the permeate pressure drop is not included in the model. Thus, some semi-empirical formulas were developed based on the above model to fit the theoretical calculations with the experimental results. In the model of Schofield et al. [26], the permeate flux of VEDCMD was calculated by introducing a semi-empirical coefficient (the ratio of the average vapor pressure at two ends of the micro-pores to the reference pressure). Based on the research of Schofield et al., Naidu et al. [27] modified the calculation procedure of VEDCMD permeate flux by introducing a dimensionless coefficient *α* (the ratio of the average pressure of two membrane surface to the reference pressure), which was similar to the semi-empirical coefficient proposed by Schofield et al. In their research, only the vapor pressure difference between the two ends of the micro-pores, or between the two surfaces of the membrane, was considered as the driving force of the transmembrane mass transfer. However, different from the DCMD, in the VEDCMD process, the mass transfer processes through the membrane pores are not only driven by the single driving force. For the diffusion pattern, the driving force should be the vapor pressure difference between the feed and permeate side, for the molecular motion is caused by the “concentration” of the water vapor molecules. Nevertheless, for the Poiseuille flow pattern, the driving force should be the total pressure difference of the two membrane sides, and the driving force caused by the vapor pressure difference is relatively small and negligible. Based on the above analyses, we propose an improved transmembrane mass transfer formula for the VEDCMD by distinguishing the contribution of the two types of driving force as shown in Equation (14). The first item at the right side of the equation represents the mass transfer caused by the diffusion of water vapor molecules, and the second item the mass transfer caused by the Poiseuille flow through the pores.

(14) $$J_{M}=K_{D}\cdot \left(p_{f,W}-p_{p,W}\right)+K_{\mathrm{PO}}\cdot \left(p_{f}-p_{p}\right)$$

where *p*_f_ (Pa) and *p*_p_ (Pa) are the total pressure of the feed channel and permeate channel. *K*_D_ is the mass transfer coefficient caused by the diffusion, and can be calculated by Equations (8)–(11) according to the *K*_n_ number.

#### 2.1.2. Heat Transfer

The heat transfer across the membrane consists of three steps:

(1) Heat is transferred from the main body of feed seawater flow to the membrane surface on the feed side.
(2) A part of the heat is taken away from the feed side and passes through the membrane by heat conduction and vaporization.
(3) The vapor condenses on the permeate side, together with the conducted heat of the membrane, raising the temperature on the permeate side.

It is reported that the heat transfer caused by convection only accounts for 0.6% of the total transferred heat hence, thus being negligible. Then, the heat transfer rate *q*_M_ (w·m^−2^) can be calculated by Equation (15) [28].

(15) $$q_{M}=q_{H}+q_{C}=J_{M}\Delta H_{V}+\frac{k_{m}}{\delta }\left(T_{f,W}-T_{p,W}\right)$$

where *q*_H_ (w·m^−2^) is the latent heat of vaporization through the membrane and *q*_C_ (w·m^−2^) is the conducted heat through the membrane, considered as heat loss. The evaporation enthalpy of water Δ*H*_V_ (kJ·kg^−1^) can be calculated by Equation (16). Since the membrane is porous, there is a gap in the membrane pores, and the thermal conductivity of the membrane *k*_m_ (J·m^−1^·k^−1^) can be calculated by Equation (17).

(16) $$\Delta H_{V}=-0.001351T_{f,W}{}^{2}-1.4461T_{f,W}+2986.5$$

(17) $$k_{m}=\varepsilon_{h}k_{a}+(1-\varepsilon_{h})k_{s}$$

where *k_a_* (J·m^−1^·k^−1^) is the thermal conductivity of air, and *k*_s_ (J·m^−1^·k^−1^) is the thermal conductivity of the membrane material.

### 2.2. CFD Model for Permeate Flux Prediction

Grounded on the proposed transmembrane transfer model of the VEDCMD, CFD simulations are carried out to predict the permeate flux of the VEDCMD driven by the low-grade heat source.

#### 2.2.1. Governing Equations

Equations (18)–(20) represent the continuity, momentum conservation, and energy conservation equations for fluid flow both in the feed and permeate channel. Equation (21) is the heat transport equation for the membrane. The component transport Equation (22) is adopted to calculate the concentration distribution of NaCl in the feed solution. For the *R*e number of the channel flow ranges more than 4000, the turbulence model should be introduced to enclose the governing equations. The standard *k*-*ε* model is adopted and the specific equations are shown as Equations (23) and (24).

(18) $$\frac{\partial \rho }{\partial t}+\nabla \cdot (\rho \vec{v})=S_{m}$$

(19) $$\frac{\partial }{\partial t}(\rho \vec{v})+\nabla \cdot (\rho \vec{v}\vec{v})=-\nabla p+\nabla \cdot \left(\overset{=}{\tau }\right)+\rho \vec{g}+\vec{F}$$

(20) $$\frac{\partial }{\partial t}(\rho E)+\nabla \cdot \left(\vec{v}\left(\rho E+p\right)\right)=\nabla \cdot \left(k_{eff}\nabla T-\sum_{j}h_{j}{\vec{J}}_{j}+\left({\overset{=}{\tau }}_{eff}\cdot \vec{v}\right)\right)+S_{h}$$

(21) $$\frac{\partial }{\partial t}(\rho E)+\nabla \cdot (\vec{v}\rho E)=\nabla \cdot (k_{m}\nabla T)$$

(22) $$\frac{\partial }{\partial t}\left(\rho Y_{i}\right)+\nabla \cdot \left(\rho \vec{v}Y_{i}\right)=-\nabla \cdot {\vec{J}}_{{}_{i}}+R_{{}_{i}}+S_{{}_{i}}$$

(23) $$\frac{\partial }{\partial t}(\rho k)+\frac{\partial }{\partial x_{i}}\left(\rho ku_{j}\right)=\frac{\partial }{\partial x_{j}}\left[\left(\mu +\frac{\mu_{t}}{\sigma_{k}}\right)\frac{\partial k}{\partial x_{j}}\right]+G_{k}+G_{b}-\rho \varepsilon -Y_{M}+S_{k}$$

(24) $$\frac{\partial }{\partial t}\left(\rho \varepsilon \right)+\frac{\partial }{\partial x_{j}}\left(\rho \varepsilon u_{j}\right)=\frac{\partial }{\partial x_{j}}\left[\left(\mu +\frac{\mu_{t}}{\sigma_{\varepsilon }}\right)\frac{\partial \varepsilon }{\partial x_{j}}\right]+\rho C_{1}S\varepsilon -\rho C_{2}\frac{\varepsilon^{2}}{k+\sqrt{v\varepsilon }}+C_{1\varepsilon }\frac{\varepsilon }{k}C_{3\varepsilon }G_{b}+S_{\varepsilon }$$

The mass source terms of the Equation (18) *S*_m_ (kg·m^−3^·s^−1^) is given by Equation (25), and the heat transfer source *S*_h_ (kW·m^−3^) is given by Equation (26). These source terms only exist in the flow at the first grid layer away from the membrane surface. In other flow areas, they are equal to 0. In the feed flow, the source term *S*_m_ is negative while in the permeate flow it is positive.

(25) $$S_{m}=\pm \frac{J_{M}}{b}$$

(26) $$S_{h}=S_{m}\cdot \Delta H_{v}$$

where *b* (mm) is the thickness of the flux at the first grid layer from the membrane surface. *k_eff_* (W·m^−1^·k^−1^) is the thermal conductivity of fluid. *h_j_* is the enthalpy of the component *j*. ${\overset{=}{\tau }}_{eff}$ (kg·m^−1^·s^−1^) is the stress tensor related to the viscous force. $\rho \vec{g}$ and $\vec{F}$ are the gravitational and external body forces, respectively. *G_k_* and *G_b_* represent the generation of turbulence kinetic energy due to the mean velocity gradients and buoyancy, respectively. *Y*_M_ is the contribution of the fluctuating dilatation in the compressible turbulence to the overall dissipation rate. *C*_1*ε*_ and *C*_2_ are constants. *σ_k_* and *σ_ε_* are the turbulent Prandtl numbers for *k* and *ε*, respectively. *S_k_* and *S_ε_* are user defined source terms.

#### 2.2.2. Simplified Geometrical Model

**Figure 1:**
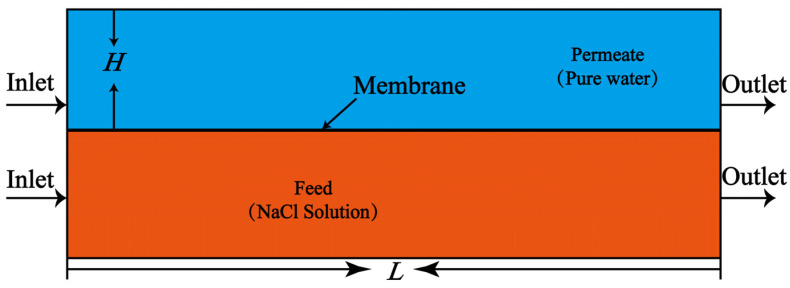
Simplified 2D geometrical model of the flat-plate VEDCMD module.

A flat-plate module is adopted for the VEDCMD simulation and its configuration is simplified to a 2D geometrical model shown in Figure 1, with the characteristic structural parameters given in Table 1.

The channel of the feed (hot seawater) and permeate side (cold freshwater) are symmetric in structure; both are 125 mm in length, 25 mm in width, and 12 mm in height. The liquid in each channel flows from the inlet to the outlet, with mass and heat transferred through a hydrophobic PVDF (polyvinylidene difluoride) membrane, which is fixed between them. The features of the membrane module are also given in Table 1.

**Figure 2:**
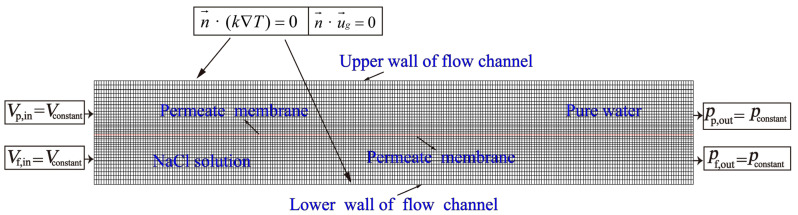
Schematic diagram of specific meshing results and boundary conditions.

The geometrical model is meshed in ICEM, and the quadrilateral structured mesh is adopted for better calculation accuracy. A local mesh densification scheme is applied for the mesh near the membrane. The height of the first layer and the growth factor are 9.7 μm and 1.01, respectively. The mesh of the feed and permeate channel flow are distributed symmetrically. The quality of the generated mesh is checked by the aspect ratio and angle, and the total grid number of the 2D model (50,196) is obtained after grid independence analysis. The mesh detail of the model is shown in Figure 2.

#### 2.2.3. Boundary Conditions

Necessary boundary conditions are set in the meshed fluid domain as shown in the Figure 2. The feed and permeate inlet are both the velocity inlet with a constant value for each simulation, and the outlets are both pressure outlets (*p*_p,out_ = *p*_f,out_ = 101,325 Pa,). As turbulence is involved, turbulence intensity and hydraulic diameter need to be calculated at the velocity inlet, and reasonable parameter values need to be set to ensure the accuracy of the simulation results. The membrane surface of both feed and permeate side are non-slip walls(*u_g_* = 0). The transmembrane mass and heat then spread to other fluid domain with the flow. The upper and lower wall of the membrane module are assumed to be adiabatic.

## 3. Experimental Setup

**Figure 3:**
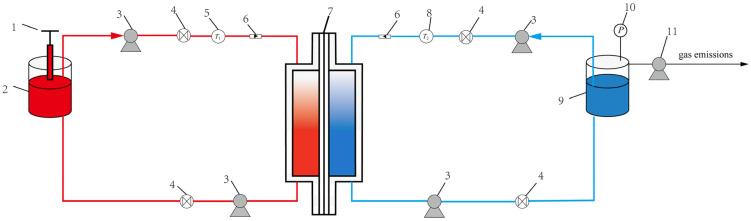
Schematic diagram of VEDCMD experiment. 1—Heater, 2—Seawater tank, 3—Micro-circulation pump, 4—Control valve, 5—Temperature sensor (seawater), 6—Liquid flow meter, 7—DCMD module, 8—Temperature sensor (freshwater), 9—Freshwater pressure stabilization tank, 10—Vacuum gauge, 11—Vacuum pump.

**Figure 4:**
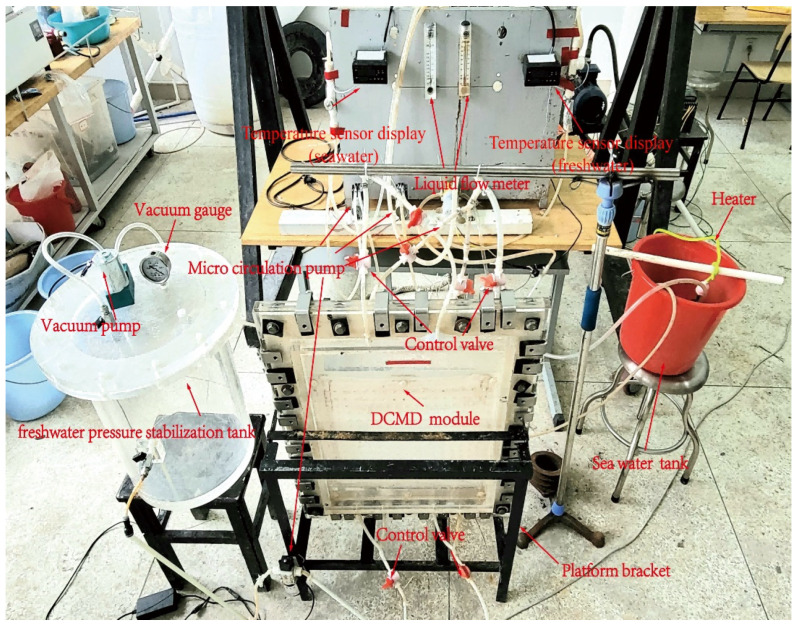
Testing platform for the VEDCMD seawater desalination.

Figure 3 and Figure 4 shows the schematic and photo of the established testing platform for the VEDCMD seawater desalination, respectively, mainly consisting of two circuits: a feed branch and a permeate branch. In the feed branch, the feed channel is connected with a hot seawater tank, a heater acting as a low-grade heat source, and a micro-circulation pump. In the permeate branch, the permeate channel is enclosed by pipelines equipped with a freshwater pressure stabilization tank with a vacuum pump, vacuum gauge, and a micro-circulation pump. Necessary temperature sensors, liquid flow meters, and control valves are mounted on the pipeline as well. The length and height of the feed and permeate channel, and the membrane characteristic parameters, are the same as those of the CFD model, and the effective membrane area is 0.136 m^2^. The module is made of PMMA (polymethyl methacrylate) so that the fluid flow in the channels and the membrane state can be observed.

Every time before the experiment is started, the heater, flow meters, temperature sensors, micro-circulation pumps, vacuum pump, vacuum gauge, and other auxiliary equipment are checked to ensure the stable operation of the equipment and instruments. All the valves are also checked to ensure the right state. The feed and permeate channel are fulfilled with seawater and freshwater, and the air inside is drained thoroughly. Meanwhile, the sealing of the membrane module and all the pipelines are inspected. The temperature of the flow at the inlet of the feed and permeate channel is maintained by adjusting the seawater volume of the seawater tank, freshwater volume of the freshwater pressure stabilization tank, and the vacuum pump.

At the beginning of the operation, seawater is injected into the seawater tank, and then heated through the heater to reach the required feed temperature. The freshwater is pumped into the freshwater pressure stabilization tank, but does not fill the tank, so that an upper air volume is formed in the tank. The vacuum pump mounted on the top of the tank is used to create a vacuum degree of the air volume, which will be conducted to the permeate flow to realize the vacuum enhancement. When the flow rate and the temperature of the both circuits become stable, the height of freshwater in the freshwater pressure stabilization tank is recorded and the timer goes to work. After 20 min, the height of the freshwater in the freshwater pressure stabilization tank is recorded again to calculate the water production rate (if necessary, a volume of cold freshwater is injected into the tank to maintain the temperature, and the induced height change will also be considered). The same operation will repeat for three times, and the average water production rate is obtained. When the operating parameter changes, the procedure above is repeated to obtain the water production rate at different conditions. The membrane flux can be calculated according to the water production rate and the membrane area, which is used to verify the results of CFD simulation based on the proposed theoretical transmembrane transfer model.

## 4. Results and Discussion

### 4.1. Experimental Verification of the Proposed Transmembrane Transfer Model

**Figure 5:**
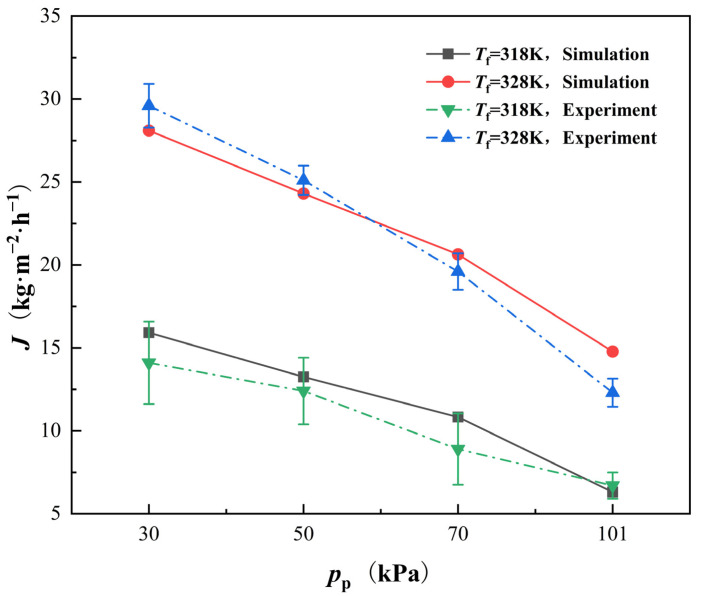
Comparison of simulating and experimental values of permeate flux of VEDCMD. *V*_f_ = 0.56 L (min), *p*_f_ = 101,325 Pa, *V*_p_ = 0.29 L (min), *T*_p_ = 303 K, and *W*_NaCl_ = 3.5%.

Figure 5 shows the comparison between the simulated membrane flux and experimental values under two different inlet temperatures of the feed channel flow. The inlet temperature of the permeate channel flow is maintained at 303 K. All above temperatures are controlled to vary within the range of ±1 K in the experiment. At any inlet temperature of the feed flow, the permeate flux increases remarkably with the vacuum degree of the feed channel flow. When the absolute pressure of the permeate flux reaches 30 kPa, the maximum permeate flux is achieved, realizing an improvement of nearly 200%, indicating the effectiveness of the VEDCMD comparing to the DCMD. The simulation results are in good agreement with the experimental data.

Meanwhile, in the model presented in the literature introducing a semi empirical coefficient, the calculation program has to be adjusted with the operating condition, because the coefficient is relevant to the operating pressure of the feed and permeate flow. It may bring tedious modifications and even mistakes in the simulation, and be unsuited for the simulations under fluctuating pressure conditions that occur fairly often in industrial practice. In contrast, all the parameters of the model proposed in this paper are independent of the operating condition, and thus the model is easier for use and has better adaptability to the fluctuating operating condition.

### 4.2. Flow Field along the Membrane Surface

The flow field along the membrane surface can hardly be obtained through experiment, but can easily be obtained from the simulated results, including the temperature and velocity of both membrane sides and the salinity of the feed side. For VEDCMD, at different pressures of the permeate side, the permeate membrane flux varies. Thus, there must be some differences between these parameter distributions, which will be revealed below (taking the case with *T*_f_ equal to 318 K as an example).

**Figure 6:**
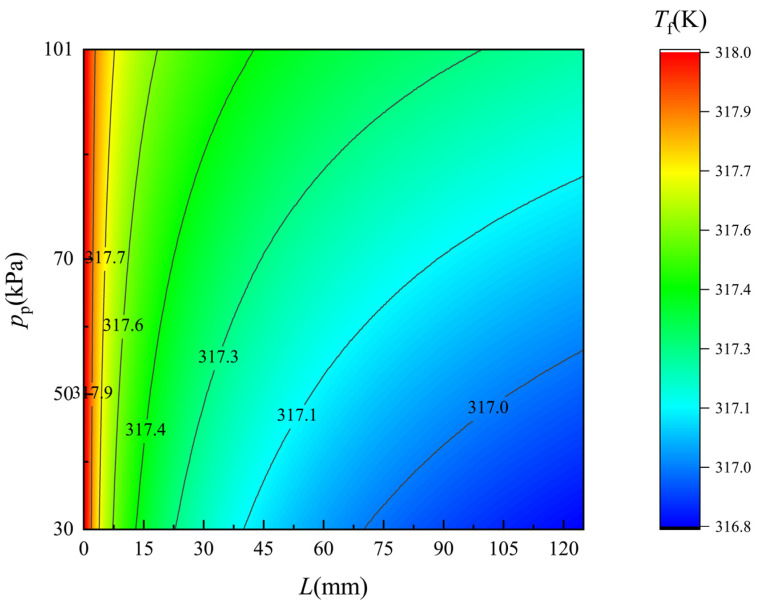
Temperature distribution of flow along the feed side membrane surface. *T*_f_ = 318 K.

**Figure 7:**
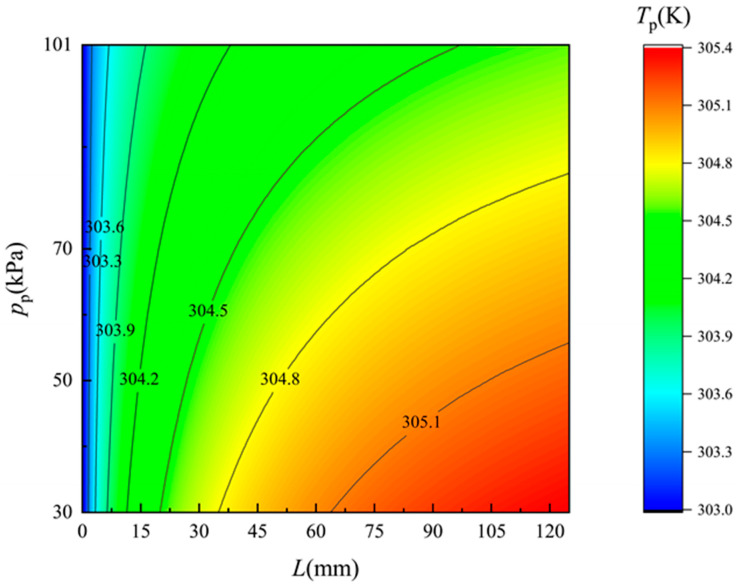
Temperature distribution of the flow along the permeate side of the membrane surface. *T*_f_ = 318 K.

Figure 6 and Figure 7 show the simulated temperature distribution of the flow along the two side of the membrane surface, respectively. The monitored flow region is 0.1 mm apart from each side of the membrane surface. On the feed side, the temperature drops obviously as the feed seawater flows along the membrane, and in contrast the temperature of the freshwater rises all along. As the pressure on the permeate side decreases, i.e., the vacuum degree of the permeate flow increases, the temperature variation become more significant due to the improvement of the permeate flux. With the increase in the permeate flux, more heat is needed for the water evaporation on the feed side and more heat will released due to the water condensation on the permeate side. Thus, the temperature distributions are in good agreement with the permeate flux variations, indicating a good consistency of mass and heat transfer of the established theoretical model and the experimental results.

**Figure 8:**
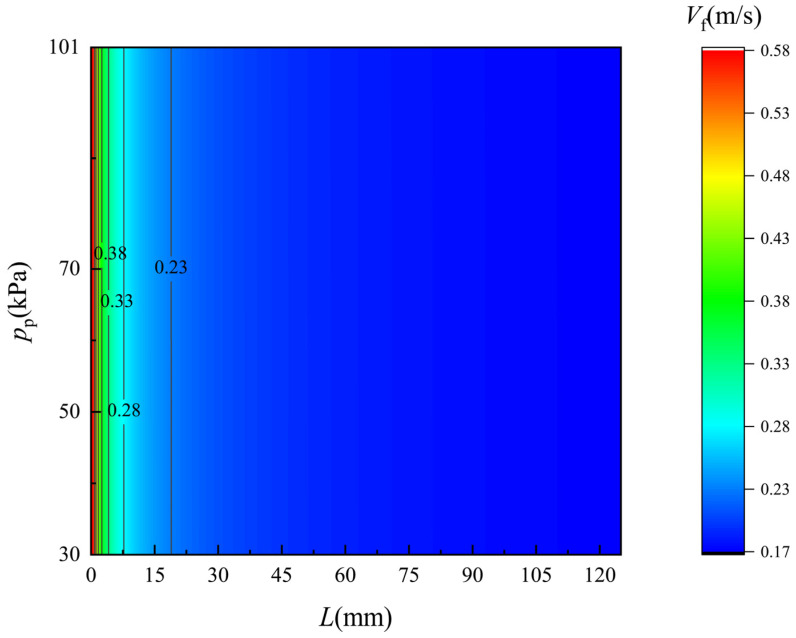
Velocity distribution of the flow along the feed side of the membrane surface. *T*_f_ = 318 K.

**Figure 9:**
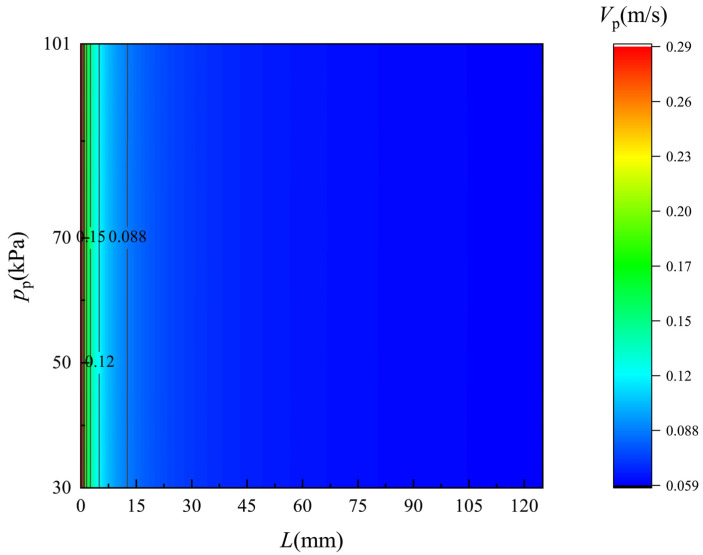
Velocity distribution of flow along the permeate side of the membrane surface. *T*_f_ = 318 K.

Figure 8 and Figure 9 show the simulated velocity distribution of the flow along the two sides of the membrane. The monitored flow region is 0.1 mm apart from the membrane surface. On the feed and permeate side, the velocity drops obviously as the seawater flows along the membrane and the trend of drops is gradually slow. As the pressure on the permeate side decreases, the velocity along the membrane surface is barely changed although the permeate flux increase. Compared to the flow flux in the feed and permeate channel, the permeate flux is really small, and thus the caused velocity variation can hardly be observed.

**Figure 10:**
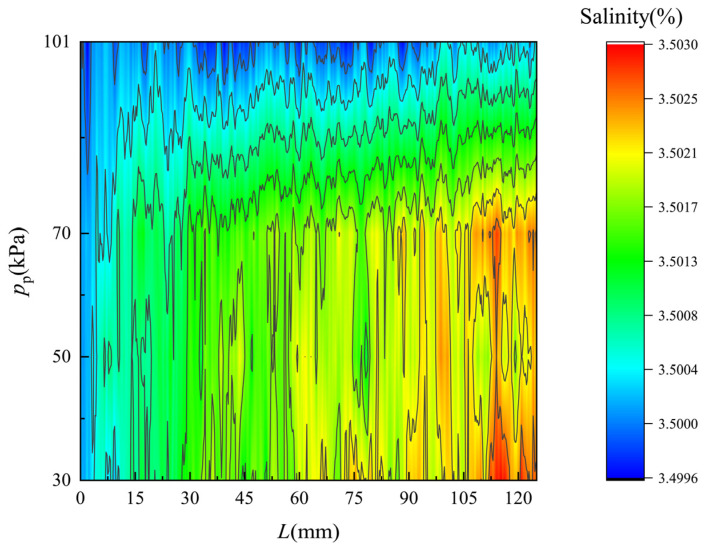
Salinity distribution of flow along the feed side of the membrane surface. *T*_f_ = 318 K.

Figure 10 shows the simulated salinity distribution of the flow along the feed side of the membrane surface. The monitored flow region is 0.1 mm apart from the membrane surface. The salinity rises obviously as the feed seawater flows along the membrane, for only freshwater vapor can pass though the pores of the membrane. As the pressure on the permeate side decreases, i.e., the vacuum degree of the permeate flow increases, the salinity variation become more significant due to the improvement of the permeate flux. Thus, the salinity distributions are in good agreement with the permeate flux variations.

## 5. Conclusions

To facilitate the permeate flux prediction of the VEDCMD, a theoretical transmembrane mass and heat transfer model is proposed with full consideration of the different driving forces of diffusion and Poiseuille flow under the vacuum enhancing condition, based on which the CFD simulations are carried out using a configuration of a flat plate module. To verity the theoretical model and the simulating results, a series of experiments are performed. By analyzing the theoretical, CFD and experimental results, the following conclusions are obtained.

(1) Compared with the model introducing a semi-empirical coefficient, all the parameters of the model proposed in this paper are independent of the operating condition, and thus the model is easier for use and has better adaptability to the fluctuating operating conditions.
(2) The simulation results based on the proposed model have good agreement with the experimental data. In the VEDCMD desalination, the permeate flux is significantly enhanced by decreasing the permeate side pressure. When the absolute pressure of the permeate side reaches 30 kPa, the permeate flux can be improved by nearly 200%.
(3) The flow fields of the flow along each side of the membrane are revealed under different pressure of the permeate side. The permeate flux rising caused by the vacuum enhancement leads to increasing temperature rise/drop and salinity, while it can hardly influence the velocity distribution.
(4) The CFD simulation results are helpful for guiding the VEDCMD operation and module structure improvement. Increasing the temperature of the feed flow and decreasing the pressure of the permeate flow can contribute to larger permeate fluxes. The length of the module should be controlled to avoid excessive heat conduction between the two sides of the membrane. In addition, the turbulence of the flow near the membrane should be enhanced for better mass transfer and smaller concentration polarization, by inserting or mounting some small obstacles near the membrane surface.

## Figures and Tables

**Table 1:** Characteristic parameters of the flat-plate module of VEDCMD.

VEDCMD Module Property	Value
Membrane material	PVDF
Porosity	0.75
Membrane nominal pore size	0.22 μm
Membrane thickness	0.12 mm
Length *L*	125 mm
Hot channel height H	12 mm
Cold channel height H	12 mm

## Data Availability

The authors declare that all data supporting the findings of this study are available within the article.

## Nomenclature

**Symbols**	
*J*	Permeation flux (kg·m^−2^·s^−1^)
*K*	Membrane distillation coefficient (kg·m^−2^·s^−1^·pa^−1^)
*p*	Pressure (Pa)
*X*_NaCl_	Molar fraction of solute
*T*	Temperature (K)
*γ*	Activity coefficient
*W*	Mass fraction
*M*	Molar mass (kg·mol^−1^)
*K*_n_	Knudsen number
*λ*	Average free path of water vapor (μm)
*d*	Diameter of membrane pore (μm)
*K*_B_	Boltzmann constant (J·mol^−1^·K^−1^)
*β*	Collision diameters(μm)
*D*_Wa_	Diffusion coefficient of water vapor (m^2^·s^−1^)
*p*_t_	Gas pressure in the pore of the membrane
*ε*_h_	Surface porosity
*τ*	Membrane tortuosity factor
*δ*	Membrane thickness (mm)
*R*	Gas constant (J·mol^−1^·k^−1^)
*r*	Membrane pore radius (μm)
*μ*	Viscosity (pa·s)
*q*	Heat flux (w·m^−2^)
*ΔH*_V_	Enthalpy of evaporation (kJ·kg^−1^)
*κ*	Thermal conductivity (J·m^−1^·k^−1^)
*ρ*	Density (kg·m^−3^)
*S*	Source term
*b*	Thickness of the first grid layer (mm)
$\overset{=}{\tau }$	Stress tensor (kg·m^−1^·s^−1^)
$\rho \vec{g}$	Gravitational body force
$\vec{F}$	External body force
*k*	Turbulent kinetic energy
*σ*	Turbulent Prandtl number
*G_b_*	Turbulence kinetic energy (buoyancy)
*G_k_*	Turbulence kinetic energy (the mean velocity gradients)
*Y*_M_	Contribution of the fluctuating dilatation
*C*_1_	constant
*C*_2_	constant
*C*_1e_	constant
*C*_3e_	constant
**Superscript**	
S	Saturation properties
**Subscripts**	
m	Membrane
f	Feed side
p	Permeate side
W	Flow channel
v	Vapor
w	Water
MD	Molecular diffusion
K	Knudsen diffusion
K-MD	Knudsen-molecular diffusion
PO	Poiseuille flow
g	Gas
D	DCMD
H	Latent heat
C	Heat conduction
*a*	Air
s	Solid
*eff*	Effective
*j*	Component
